# Tumor Microenvironment of Metastasis (TMEM) Doorways Are Restricted to the Blood Vessel Endothelium in Both Primary Breast Cancers and Their Lymph Node Metastases

**DOI:** 10.3390/cancers11101507

**Published:** 2019-10-08

**Authors:** Paula S. Ginter, George S. Karagiannis, David Entenberg, Yu Lin, John Condeelis, Joan G. Jones, Maja H. Oktay

**Affiliations:** 1 Department of Pathology and Laboratory Medicine, Weill Cornell Medicine, New York, NY 10065, USA; psg9003@med.cornell.edu; 2 Department of Anatomy and Structural Biology, Einstein College of Medicine/Montefiore Medical Center, Bronx, NY 10461, USA; georgios.karagiannis@einstein.yu.edu (G.S.K.); david.entenberg@einstein.yu.edu (D.E.); yu.lin@einstein.yu.edu (Y.L.); john.condeelis@einstein.yu.edu (J.C.); 3 Gruss-Lipper Biophotonics Center, Einstein College of Medicine/Montefiore Medical Center, Bronx, NY 10461, USA; 4 Integrated Imaging Program, Einstein College of Medicine/Montefiore Medical Center, Bronx, NY 10461, USA; 5 Department of Surgery, Einstein College of Medicine/Montefiore Medical Center, Bronx, NY 10461, USA; 6 Department of Pathology, Einstein College of Medicine/Montefiore Medical Center, Bronx, NY 10461, USA; 7 Department of Epidemiology and Population Health, Einstein College of Medicine/Montefiore Medical Center, Bronx, NY 10461, USA

**Keywords:** breast cancer, lymphatic vessel, lymph node, blood vessel, tumor microenvironment of metastasis (TMEM)

## Abstract

Cancer cells metastasize from primary tumors to regional lymph nodes and distant sites via the lymphatic and blood vascular systems, respectively. Our prior work has demonstrated that in primary breast tumors, cancer cells utilize a three-cell complex (known as tumor microenvironment of metastasis, or TMEM) composed of a perivascular macrophage, a tumor cell expressing high levels of the actin-regulatory protein mammalian enabled (Mena), and an endothelial cell as functional “doorways” for hematogenous dissemination. Here, we studied a well-annotated case–control cohort of human invasive ductal carcinoma of the breast and metastatic lymph nodes from a separate breast cancer cohort. We demonstrate that in primary breast tumors, blood vessels are always present within tumor cell nests (TCNs) and tumor-associated stroma (TAS), while lymphatic vessels are only occasionally present in TCN and TAS. Furthermore, TMEM doorways not only exist in primary tumors as previously reported but also in lymph node metastases. In addition, we show that TMEM intravasation doorways are restricted to the blood vascular endothelium in both primary tumors and lymph node metastases, suggesting that breast cancer dissemination to distant sites from both primary tumors and metastatic foci in lymph nodes occurs hematogenously at TMEM doorways. TMEMs are very rarely detected at lymphatic vessels and do not confer clinical prognostic significance, indicating they are not participants in TMEM-associated hematogenous dissemination. These findings are consistent with recent observations that hematogenous dissemination from lymph nodes occurs via blood vessels.

## 1. Introduction

In breast cancer, tumor cells may disseminate to lymph nodes and more distant organs, but the mechanism of dissemination and the subsequent risk of mortality resulting from these cells may not be the same. Over the last 15 years, lymphatic spread has been studied most extensively in breast cancer and melanoma, but the direct involvement of lymphatic vessels in systemic tumor cell dissemination from primary tumors has not been conclusively demonstrated [1]. Furthermore, the attitude regarding the significance of lymph node metastases in breast cancer is changing in light of recent reports that full axillary lymph node dissection does not improve overall or disease-free survival when compared to sentinel lymph node dissection alone in cases of sentinel node involvement and significance of micrometastases in lymph nodes has been questioned as detection of these occult lymph node metastases do not provide a great clinical benefit to patients [2,3]. Nevertheless, the number of lymph nodes (LNs) involved with metastatic tumor is undoubtedly one of the most important prognostic factors most likely indicating the overall ability of cancer cells to disseminate. 

During cancer progression, highly migratory tumor cells disseminate to regional lymph nodes and/or distant organs, where they may seed secondary metastases in a multistep biological process, collectively known as the metastatic cascade [4]. While metastasis to the regional lymph nodes is considered localized disease, and is potentially curable, dissemination to distant organs is deemed true metastatic disease and is associated with high mortality. The metastatic cascade most often begins with the detachment of single tumor cells from multicellular tumor cell nests following epithelial-to-mesenchymal transition (EMT) [5,6]. To disseminate hematogenously, these cells migrate towards intratumoral blood vessels and intravasate using specialized blood vessel doorways called the tumor microenvironment of metastasis (TMEM) [7]. TMEM doorways are composed of a perivascular macrophage, a tumor cell highly expressing the actin-regulatory protein mammalian enabled (Mena), and a vascular endothelial cell, all in direct physical contact with each other [8,9]. While each component of TMEM is critical in the process of this hematogenous dissemination, it has been elucidated in prior clinical studies that the density of TMEM doorways in primary breast tumors is prognostic of distant metastasis, independently of lymph node status and other currently used prognosticators [10,11,12].

Given that lymph node status and lymphovascular invasion within the primary tumor are both predictors of metastatic risk [13,14], we sought to determine whether the lymphatic endothelium in primary tumors participates in assembling an intravasation-competent lymphatic vessel TMEM (LV-TMEM) doorway analogous to the one observed for blood vessel TMEM (BV-TMEM) doorways. Since recent studies in animal models indicate that tumor cells can utilize the blood vasculature to further disseminate systemically from the lymph nodes [15,16], we examined if TMEM doorways were also present in metastatic foci in the lymph nodes and evaluated whether these doorways were LV-TMEM or BV-TMEM.

## 2. Results

### 2.1. Distribution of BVs (Blood Vessels) and LVs (Lymphatic Vessels) in Healthy Breast and Neoplastic Breast Tissue 

**Figure 1:**
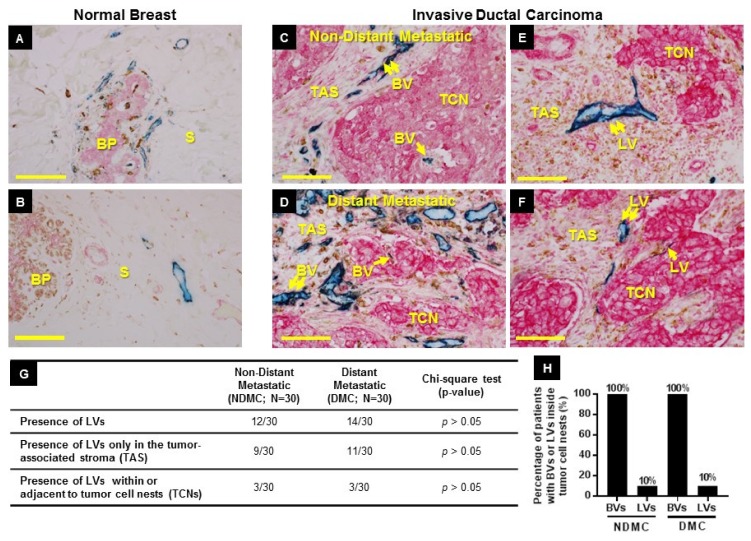
Location of blood vessels (BVs) and lymphatic vessels (LVs) in normal and neoplastic breast tissue. (**A**,**B**) Normal breast. (**A**) BVs (blue, CD31) are seen in close proximity to the breast glandular parenchyma (BP) as well as in the adjacent connective tissue stroma (S). (**B**) LVs (blue, D2-40) are seen in the stroma (S). (**C**–**F**) BVs and LVs in primary tumors of patients with metastatic and non-metastatic breast cancer. (**C**,**D**) In all non-distant metastatic (**C**) and distant metastatic (**D**) cases, BVs are found both in tumor cell nests (TCNs) (single arrow) and in tumor-associated stroma (TAS) (double arrow). (**E**,**F**) In some cases (**E**), LVs are found only in TAS (double arrow), and only rarely (F) are LVs found both in TAS (double arrow) and in TCNs (single arrow). Scale bars (**A**–**F**) = 90 µm. (**G**) Frequency of LV presence in the primary tumors of non-distant metastatic and distant metastatic patient cohorts. (**H**) Percentage (%) of patients with BVs or LVs inside tumor cell nests in the primary tumors of non-distant metastatic and metastatic patient cohort. See Table 2 for panels **C**–**G** raw data.

To examine the status of the lymphatic and blood vasculature in our breast cancer patient cohorts, we first evaluated the localization of BVs and LVs in relation to benign breast stroma and benign breast glandular parenchyma (Figure 1A,B), and then assessed the localization of BVs and LVs in relation to invasive tumor cell nests (TCNs) and the adjacent tumor-associated stroma (TAS) (Figure 1C–F). A total of 60 primary tumors from a matched population of breast cancer patients, half of whom developed distant metastasis (distant metastatic cohort (DMC), *n* = 30) and half of whom did not (non-distant metastatic cohort (NDMC), *n* = 30), were examined using a previously described TMEM triple-stain immunohistochemistry (IHC) assay (BV-TMEM), as well as a modified TMEM triple-stain IHC assay (see TMEM Immunohistochemistry in Materials and Methods) specific for lymphatic vessels (LV-TMEM). Benign human breast tissue was used as a control. In contrast to BVs that occurred in both the benign breast glandular parenchyma and the benign breast stroma (Figure 1A), LVs were exclusively present in the benign breast stroma (Figure 1B). 

In primary breast tumors, BVs were interspersed in both the TCNs and the TAS in all cases (Figure 1C,D), and 100% of patients had BVs in the TCNs (Figure 1H). On the contrary, only 12/30 NDMC cases and 14/30 DMC cases had LVs in TCNs and TAS (Figure 1E,F,G), and the density was low, typically <1 per 10 high-power fields (HPFs) on average. Moreover, the presence of any LVs within TCNs was observed in only 10% of cases (Figure 1H). The presence of LVs in either TAS or within TCNs did not significantly correlate (*p* > 0.05) with distant metastasis in this patient cohort (Figure 1G). 

While BVs were universally present in all primary tumors examined, LVs were found only in a subset of cases. Therefore, we evaluated if the presence or absence of LVs was associated with a particular breast cancer subtype and found no correlation (*p* > 0.05; Table 1A,B). Since only a subset of breast cancer patients had LVs in their tumors, we next classified the distribution of LVs within these tumors into two histological patterns—(i) LVs in TAS, and (ii) LVs within or directly adjacent to TCNs—and evaluated the distribution of these two patterns among different breast cancer subtypes. Neither of the two histological patterns was associated with a particular breast cancer subtype (*p* > 0.05; Table 1C). The detailed analysis of the presence or absence of BVs and LVs, their location within NDMC and DMC and relationship to breast cancer subtype is shown in Table 2. 

In summary, these data indicate that BVs are universally present in both TAS and TCNs in all breast cancer subtypes while LVs are present in either location in less than 50% of cases and are found in TCNs in only 10% of cases. 

### 2.2. Quantitation of BV-TMEM and LV-TMEM in Primary Breast Tumors

**Figure 2:**
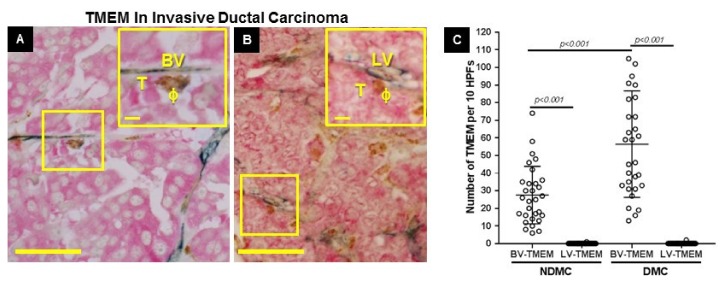
Blood vessel-tumor microenvironment of metastasis (BV-TMEM) are common in primary breast tumors and lymphatic vessel (LV-TMEM) are very rare. (**A**) BV-TMEM is a tripartite structure composed of a BV (blue, CD31), a perivascular macrophage (ϕ, brown, CD68), and a Mena-overexpressing tumor cell (T, pink, pan-Mena), all in direct contact. (**B**) LV-TMEM is a tripartite structure composed of an LV (blue, D2-40), a perivascular macrophage (ϕ, brown, CD68), and a Mena-overexpressing tumor cell (T, pink, pan-Mena), all in direct contact. Examples of TMEM locations are specified in yellow boxes at both low and high magnification (insets). Scale bars = 80 µm. Inset scale bars = 10 µm. (**C**) Quantification of BV-TMEM and LV-TMEM in the non-distant metastatic (NDMC) and distant metastatic human (DMC) patient cohorts (in all comparisons *p* < 0.001). See Table 3 for raw data.

In both distant metastatic (DMC) and non-distant metastatic (NDMC) patient cohorts, BV-TMEM were found in all cases (Figure 2A, Table 3). However, given the overall lack of LVs in contact with TCNs, LV-TMEM were identified in only two cases, and in these two cases, were only rarely observed (*n* = 3 in total) (Figure 2B, Table 3). As expected, the BV-TMEM scores in the DMC cohort were significantly higher than in the NDMC (*p* < 0.001; Figure 2C), whereas for LV-TMEM, there was no significant difference between the two cohorts. In addition, neither the presence of BV-TMEM nor of LV-TMEM were associated with any particular breast cancer subtype (*p* > 0.05; Table 4). Overall, these data indicate that the TMEM-mediated mechanism of cancer cell dissemination from the primary tumors is restricted to blood vasculature. Lymphatic vessels were not involved.

### 2.3. Distribution of BVs and LVs in Established Lymph Node Metastases

**Figure 3:**
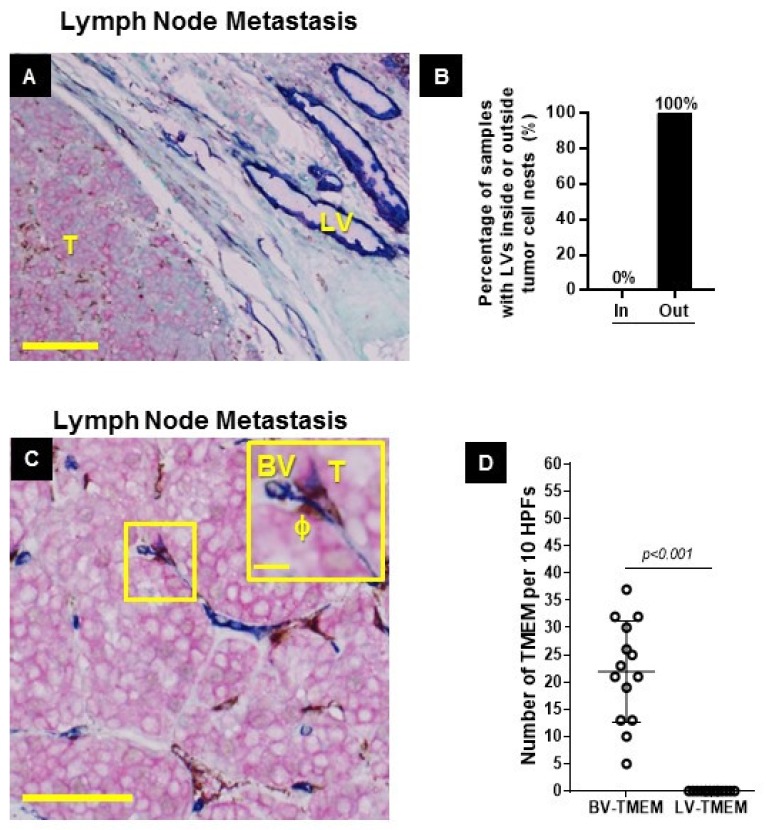
Blood vessel-tumor microenvironment of metastasis (BV-TMEM) are common in lymph node metastases and lymphatic vessel TMEM (LV-TMEM) are absent. (**A**) Metastatic tumor mass (T) in a breast cancer patient lymph node from a separate breast cancer cohort. The tumor mass, on the left, contains no LVs. LVs are only seen outside the tumor mass on the right (blue). (**A**,**C**) Pink = tumor cells (stained for pan-Mena), brown = macrophages (stained for CD68), blue = blood or lymphatic vessels (stained for D2-40 and CD31, respectively. Scale bar = 100 µm. (**B**) Frequency (%) of LVs inside or outside the metastatic tumor mass in lymph nodes. (**C**) BV-TMEM in a lymph node of a breast cancer patient with a metastatic tumor mass. No LV-TMEM were identified within metastatic tumor masses. Scale bar = 120 µm. Inset scale bar = 20 µm. (**D**) Distribution of scores for BV-TMEM and LV-TMEM in the lymph nodes with tumor masses. The differences between the BV-TMEM and LV-TMEM scores in each cohort are significant (*p* < 0.001). See Table 5 for raw data.

Stains for BV-TMEM and LV-TMEM allowed us to determine the distribution of BVs and LVs in established lymph node metastases from a separate breast cancer cohort. BVs were present both within and outside established metastatic tumor masses in lymph nodes. LVs, however, were completely absent within the metastatic tumor foci. LVs were only present outside the metastatic tumor mass (Figure 3A,B).

### 2.4. BV-TMEM are Present in Established Lymph Node Metastases, whereas LV-TMEM are not Present in Established Lymph Node Metastases

We next evaluated the presence of TMEM doorways in established lymph node metastases. Given the lack of LVs in established lymph node metastases, the absence of LV-TMEM was expected. We did find that TMEM doorways are exclusively associated with BVs (Figure 3C,D, Table 5). In conclusion, these data indicate that once metastatic tumors are established in regional lymph nodes, tumor cells can then prompt the de novo assembly of conventional blood vessel-based TMEM doorways. LV-TMEM were not present and, hence, are not involved in TMEM-mediated cancer cell dissemination from the lymph node metastatic foci. 

## 3. Discussion

Since breast cancer mortality is predominately due to metastases to distant organs, it is imperative to elucidate the critical steps involved in distant metastasis. Two types of vasculature, blood and lymphatic, have been implicated in breast cancer dissemination to distant sites. However, which one of the two vascular systems is the major transport route of cancer cells to distant organs has been a matter of debate [17]. 

We previously showed that breast cancer cells metastasize from primary tumors to distant sites via a hematogenous route in which single tumor cells intravasate at specialized blood vessel intravasation doorways known as the tumor microenvironment of metastasis (TMEM) [7,8,18]. In this study, we aimed to demonstrate whether LVs (lymphatic vessels) participate in the formation of an analogous type of doorway (LV-TMEM), either in primary breast cancers or in their lymph node metastases or in both. 

We observed here that LVs are sparse within primary breast tumors and are present primarily at the tumor–stroma interface, as also previously reported by other groups [19]. Likewise, we observed only rare LV-TMEM doorways within primary tumors, and found no relationship between the density of LV-TMEM doorways and metastatic outcome. In addition, we identified LVs in the peritumoral stroma, with and without tumor emboli. These LVs are plausible conduits for the delivery of tumor cells to regional lymph nodes. Indeed, it has been well documented that lymph node metastases can occur as a result of lymphatic channel invasion around the primary tumor, which on histologic evaluation, is frequently identified beyond the invasive tumor borders and in the peritumoral stroma [19,20]. 

When assessing intratumoral lymphatic and blood microvessel density, studies have shown that blood vessel density is consistently increased in invasive breast carcinomas [21], whereas lymphatics may be absent or present [22,23,24,25,26]. Aligned with these observations, we observed that LVs, as well as LV-TMEM doorways, are sparse in the primary tumors and absent from established metastatic tumor masses, while BV-TMEM doorways are present in both primary tumors and established lymph node metastases. The finding of BV-TMEM doorways in lymph node metastases is consistent with our prior findings of TMEM doorways in other non-primary tumor sites, such as metastatic nodules in the lungs [27], where we have demonstrated by intravital imaging that TMEM doorways are portals for cancer cell intravasation. Thus, it is likely that BV-TMEM doorways serve as portals for cancer cell dissemination at all sites where tumor nodules are found, and cancer cell metastasis can perpetuate from all sites.

The presence of BV-TMEM in lymph node metastases is consistent with recent reports of hematogenous route of cancer cell seeding to distant sites from lymph node metastases in breast cancer [15,16]. These new discoveries challenge the historically established belief that after cancer cells reach lymph nodes via lymphatic vessels, they may exit the lymph nodes via the efferent lymphatic vessels and enter the systemic circulation using the venous system [28]. 

Although the number of positive lymph nodes is the most significant prognostic indicator for many epithelial cancers, including breast [29], the biological reasons behind this observation are not well understood. Since lymph node metastases often precede systemic disease, a common belief has been that positive lymph nodes give rise to distant metastases [30,31,32]. Indeed, the removal of positive lymph nodes in an attempt to improve patient survival has been standard of care for more than a century [30,31]. However, clinical studies showed that the removal of positive lymph nodes does not prolong overall survival of patients with breast cancer [2,33]. Similarly, in melanoma patients, lymph node dissection does not increase melanoma-specific survival among patients with lymph node metastasis [34].

Collectively, these observations indicate that cancer cell dissemination from lymph node metastases is an indicator of systemic disease spread that requires a systemic treatment approach. Moreover, the functionality of BV-TMEM doorways as portals for cancer cell dissemination to distant sites from lymph node metastases needs to be determined in preclinical models of breast cancer.

## 4. Materials and Methods 

### 4.1. Patient Samples and Information

Patient samples included formalin-fixed paraffin-embedded (FFPE) tissue from both primary tumors and lymph nodes with metastatic tumor. The primary tumor samples (*n* = 60) were from a case–control study of 60 patients assembled at NewYork-Presbyterian Hospital/Weill Cornell Medicine that was previously analyzed for BV-TMEM [9]. The cohort consisted of 30 matched pairs—a distant metastasis cohort (DMC) (*n* = 30) and a non-distant metastasis cohort (NDMC) (*n* = 30)—that were matched for tumor grade, tumor size, presence or absence of lymph node metastasis, and hormone receptor status. This cohort was composed of 42 hormone receptor (HR)-positive and either human epidermal growth factor receptor 2 (HER2)-positive or HER2-negative tumors (defined as luminal); 9 HER2-positive, HR-negative tumors (defined as HER2+), and 9 HR- and HER2-negative tumors (defined as triple negative breast cancers (TNBC)). Metastatic lymph node samples (*n* = 14) were from a separate breast cancer cohort assembled at Montefiore Medical Center [35]. All these samples were of the HR-positive HER2-negative breast cancer subtype. All described studies were performed under institutional review board (IRB# 2017-8158; approved on 4th October 2017) approval from the respective institutions.

### 4.2. TMEM Immunohistochemistry

Slides from primary tumor and lymph node metastases were stained with a lymphatic vessel-based immunohistochemistry (IHC) protocol analogous to the blood vessel-based TMEM stain. The human lymphatic protocol used D2-40 (1:50 dilution; Covance Signet Antibodies, Princeton, NY, USA) to detect lymphatic endothelial cells while the macrophage (CD68) and pan-Mena markers remained the same [10,11]. The lymph nodes with metastases were stained with the standard BV-TMEM protocol as previously reported [10,11]. 

The initial evaluation of primary tumors included an assessment of the presence and distribution of lymphatic vessels in normal and neoplastic breast tissue. In normal breast tissue, lymphatics were classified as to whether they were 1) present, 2) surrounded by stroma, or 3) within breast glandular parenchyma. In neoplastic breast tissue, lymphatics were classified as to whether they were 1) present, 2) surrounded by tumor-associated stroma (TAS), or 3) within tumor cell nests (TCNs). By definition, a BV-TMEM requires the direct contact between a blood vessel endothelial cell, a macrophage, and a Mena-expressing tumor cell. Similarly, a LV-TMEM requires the direct contact between a lymphatic endothelial cell (blue), a macrophage (brown), and a Mena-expressing tumor cell (red). Therefore, only when lymphatics were present, and found in association with TCNs, was there the possibility of the formation of an LV-TMEM. In cases where intratumoral LVs were present, 10 separate digital photographs at 400× magnification were taken in the areas of highest lymphatic and tumor cell density (total area evaluated = 1.35 mm^2^) and the number of LV-TMEM was quantified. 

For the assessment of the primary tumors, 50% (*n* = 30) of the cases were randomly assigned and assessed independently by two pathologists. The concordance on assessing the presence or absence of lymphatics within a tumor was 100%, as was the assessment of whether lymphatics within a tumor were surrounded by stroma. Therefore, the remaining cases were assessed by one pathologist for these parameters. The incidence of lymphatics within TCNs and identification of possible LV-TMEM was so uncommon that all cases were assessed by two pathologists to ensure complete concordance. Both pathologists were blinded to the clinical outcome. After tabulating the number of LV-TMEM for all samples, the cases were unblinded with regard to their metastatic status and the differences in LV-TMEM density and lymphatic distribution between metastatic and non-metastatic cohorts were evaluated. BV-TMEM scores and LV-TMEM scores were then compared based on metastatic status.

LV-TMEM and BV-TMEM scores were evaluated and compared by one pathologist in a similar manner in the 14 metastatic lymph node samples. The scores for both BV-TMEM and LV-TMEM were reported as number of TMEM doorways per 10 high-power fields (HPFs), as described previously [5,8,9].

### 4.3. Statistical Analysis

The Chi-Square test was used to evaluate if the presence or distribution of LVs within the primary tumor correlated with metastatic outcome in the human cohort. The association of LVs, BVs, and their respective TMEM structures with breast cancer subtypes in the primary tumor cohort was assessed using Chi-Square test, or Fisher’s exact test if the number of subjects was fewer than 5. Comparisons between BV-TMEM and LV-TMEM scores in the distant metastatic (DMC) and non-distant metastatic (NDMC) cohorts were assessed using the Mann–Whitney U-test. All graphs were plotted with their mean and standard deviation (SD). GraphPad Prism 7.01 (San Diego, CA, USA) was used for both graphing and statistical hypothesis testing. 

## 5. Conclusions

We showed that LV-based doorways analogous to BV-TMEM are essentially nonexistent in primary breast cancers and are completely absent from their lymph node metastases, while BV-TMEM are present at both anatomical sites. These findings suggest that breast cancers use a common, BV-TMEM based dissemination mechanism that leads to distant metastases regardless of the anatomical location of the tumor.

## Figures and Tables

**Table 1:** Association of LVs and BVs with breast cancer subtypes in a patient cohort.

A				
	# of Cases	BVs Present	BVs Absent	Chi-Square
Luminal	42	42 (100%)	0 (0%)	*p* > 0.05
HER2+	9	9 (100%)	0 (0%)	
TNBC	9	9 (100%)	0 (0%)	
**B**				
	# of Cases	LVs Present	LVs Absent	Chi-square
Luminal	42	16 (38%)	26 (62%)	*p* > 0.05
HER2+	9	5 (56%)	4 (44%)	
TNBC	9	5 (56%)	4 (44%)	
**C**				
	# of Cases with LVs Present	Histological Pattern i	Histological Pattern ii	Chi-Square
Luminal	16	14 (87.5%)	2 (12.5%)	*p* > 0.05
HER2+	5	3 (60%)	2 (40%)	
TNBC	5	3 (60%)	2 (40%)	

Fisher’s exact test was used instead of Chi-Square, if expected value in any cell was <5. Abbreviations: #, number; BV, blood vessel; LV, lymphatic vessel; Luminal, luminal breast cancer; HER2, human epidermal growth factor receptor 2-enriched breast cancer; TNBC, triple-negative breast cancer; histological pattern “i” = LVs in the tumor-associated stroma (TAS) away from tumor cells; histological pattern “ii” = LVs within or directly adjacent to tumor cell nests (TCNs).

**Table 2:** Presence or absence of BVs and LVs, and their location with regard to tumor cell nests and tumor-associated stroma or both.

Non Distant Metastatic Cohort						Distant Metastatic Cohort					
Patient ID	Breast Cancer Subtype	Presence of Vessels		Location of Vessels		Patient ID	Breast Cancer Subtype	Presence of Vessels		Location of Vessels	
		BV	LV	BV	LV			BV	LV	BV	LV
1	L	Y	N	T+S	-	31	L	Y	N	T+S	-
2	L	Y	N	T+S	-	32	L	Y	N	T+S	-
3	L	Y	N	T+S	-	33	L	Y	N	T+S	-
4	L	Y	N	T+S	-	34	L	Y	N	T+S	-
5	L	Y	Y	T+S	S	35	L	Y	N	T+S	-
6	L	Y	N	T+S	-	36	L	Y	Y	T+S	S
7	TNBC	Y	N	T+S	-	37	TNBC	Y	N	T+S	-
8	HER2	Y	N	T+S	-	38	HER2	Y	N	T+S	-
9	L	Y	N	T+S	-	39	L	Y	Y	T+S	S
10	HER2	Y	Y	T+S	T+S	40	HER2	Y	N	T+S	-
11	L	Y	Y	T+S	S	41	L	Y	N	T+S	-
12	L	Y	N	T+S	-	42	L	Y	N	T+S	-
13	HER2	Y	Y	T+S	S	43	TNBC	Y	Y	T+S	S
14	L	Y	Y	T+S	S	44	L	Y	N	T+S	-
15	TNBC	Y	N	T+S	-	45	TNBC	Y	Y	T+S	T+S
16	TNBC	Y	Y	T+S	S	46	HER2	Y	N	T+S	-
17	HER2	Y	Y	T+S	S	47	TNBC	Y	Y	T+S	T+S
18	TNBC	Y	N	T+S	-	48	TNBC	Y	Y	T+S	S
19	L	Y	N	T+S	-	49	L	Y	N	T+S	-
20	HER2	Y	Y	T+S	T+S	50	HER2	Y	Y	T+S	S
21	L	Y	N	T+S	-	51	L	Y	N	T+S	-
22	L	Y	Y	T+S	S	52	L	Y	Y	T+S	S
23	L	Y	Y	T+S	S	53	L	Y	N	T+S	-
24	L	Y	Y	T+S	S	54	L	Y	Y	T+S	T+S
25	L	Y	N	T+S	-	55	L	Y	Y	T+S	S
26	L	Y	Y	T+S	T+S	56	L	Y	Y	T+S	S
27	L	Y	N	T+S	-	57	L	Y	Y	T+S	S
28	L	Y	N	T+S	-	58	L	Y	N	T+S	-
29	L	Y	N	T+S	-	59	L	Y	Y	T+S	S
30	L	Y	N	T+S	-	60	L	Y	Y	T+S	S

Abbreviations: BV, blood vessel; LV, lymphatic vessel; Y, Yes; N, No; L, luminal breast cancer; HER2, human epidermal growth factor receptor 2-enriched breast cancer; TNBC, triple-negative breast cancer; T, vessels in contact with tumor cell nests (TCNs); S, vessels surrounded by tumor-associated stroma (TAS).

**Table 3:** BV-TMEM and LV-TMEM scores in the non-distant metastatic and distant metastatic patient cohort.

Non Metastatic Cohort				Metastatic Cohort			
Patient ID	Breast cancer Subtype	BV-TMEM	LV-TMEM	Patient ID	Breast cancer Subtype	BV-TMEM	LV-TMEM
1	L	12	0	31	L	13	0
2	L	10	0	32	L	31	0
3	L	8	0	33	L	31	0
4	L	7	0	34	L	16	0
5	L	35	0	35	L	33	0
6	L	20	0	36	L	19	0
7	TNBC	51	0	37	TNBC	65	0
8	HER2	14	0	38	HER2	63	0
9	L	42	0	39	L	39	0
10	HER2	16	1	40	HER2	46	0
11	L	34	0	41	L	38	0
12	L	34	0	42	L	72	0
13	HER2	74	0	43	TNBC	61	0
14	L	17	0	44	L	90	0
15	TNBC	17	0	45	TNBC	33	2
16	TNBC	25	0	46	HER2	20	0
17	HER2	13	0	47	TNBC	35	0
18	TNBC	46	0	48	TNBC	45	0
19	L	16	0	49	L	95	0
20	HER2	30	0	50	HER2	91	0
21	L	32	0	51	L	40	0
22	L	6	0	52	L	105	0
23	L	36	0	53	L	59	0
24	L	48	0	54	L	59	0
25	L	27	0	55	L	27	0
26	L	24	0	56	L	128	0
27	L	18	0	57	L	72	0
28	L	26	0	58	L	102	0
29	L	58	0	59	L	82	0
30	L	30	0	60	L	83	0

Abbreviations: BV, blood vessel; LV, lymphatic vessel; TMEM, tumor microenvironment of metastasis; L, luminal breast cancer; HER2, human epidermal growth factor receptor 2-enriched breast cancer; TNBC, triple-negative breast cancer.

**Table 4:** Association of BV-TMEM and LV-TMEM with breast cancer subtypes in a patient cohort.

	# Of Cases	# Of Cases with BV-TMEM (%)	# Of Cases with LV- TMEM (%)	Chi-Square
Luminal	42	42 (100%)	0 (0%)	*p* > 0.05
HER2+	9	9 (100%)	1 (9%)	
TNBC	9	9 (100%)	1 (9%)	

Fisher’s exact test was used instead of Chi-Square, if expected value in any cell was <5. Abbreviations: #, number; BV, blood vessel; LV, lymphatic vessel; TMEM, tumor microenvironment of metastasis; Luminal, luminal breast cancer; HER2, human epidermal growth factor receptor 2-enriched breast cancer; TNBC, triple-negative breast cancer.

**Table 5:** BV-TMEM and LV-TMEM scores in lymph node metastasis.

Sample ID	BV-TMEM	LV-TMEM
1	21	0
2	13	0
3	23	0
4	13	0
5	19	0
6	37	0
7	21	0
8	5	0
9	10	0
10	30	0
11	32	0
12	32	0
13	25	0
14	26	0

Abbreviations: BV, blood vessel; LV, lymphatic vessel; TMEM, tumor microenvironment of metastasis.
